# Effects of omecamtiv mecarbil on the contractile properties of skinned porcine left atrial and ventricular muscles

**DOI:** 10.3389/fphys.2022.947206

**Published:** 2022-08-23

**Authors:** Tomohiro Nakanishi, Kotaro Oyama, Hiroyuki Tanaka, Fuyu Kobirumaki-Shimozawa, Shuya Ishii, Takako Terui, Shin’ichi Ishiwata, Norio Fukuda

**Affiliations:** ^1^ Department of Cell Physiology, The Jikei University School of Medicine, Tokyo, Japan; ^2^ Department of Anesthesiology, The Jikei University School of Medicine, Tokyo, Japan; ^3^ Quantum Beam Science Research Directorate, National Institutes for Quantum Science and Technology, Gunma, Japan; ^4^ Laboratory of Marine Biotechnology and Microbiology, Hokkaido University, Hakodate, Japan; ^5^ Department of Physics, Faculty of Science and Engineering, Waseda University, Tokyo, Japan

**Keywords:** Ca^2+^ sensitivity, contractility, myocardium, sarcomere, thin filament

## Abstract

Omecamtiv mecarbil (OM) is a novel inotropic agent for heart failure with systolic dysfunction. OM prolongs the actomyosin attachment duration, which enhances thin filament cooperative activation and accordingly promotes the binding of neighboring myosin to actin. In the present study, we investigated the effects of OM on the steady-state contractile properties in skinned porcine left ventricular (PLV) and atrial (PLA) muscles. OM increased Ca^2+^ sensitivity in a concentration-dependent manner in PLV, by left shifting the mid-point (pCa_50_) of the force-pCa curve (ΔpCa_50_) by ∼0.16 and ∼0.33 pCa units at 0.5 and 1.0 μM, respectively. The Ca^2+^-sensitizing effect was likewise observed in PLA, but less pronounced with ΔpCa_50_ values of ∼0.08 and ∼0.22 pCa units at 0.5 and 1.0 μM, respectively. The Ca^2+^-sensitizing effect of OM (1.0 μM) was attenuated under enhanced thin filament cooperative activation in both PLV and PLA; this attenuation occurred directly via treatment with fast skeletal troponin (ΔpCa_50_: ∼0.16 and ∼0.10 pCa units in PLV and PLA, respectively) and indirectly by increasing the number of strongly bound cross-bridges in the presence of 3 mM MgADP (ΔpCa_50_: ∼0.21 and ∼0.08 pCa units in PLV and PLA, respectively). It is likely that this attenuation of the Ca^2+^-sensitizing effect of OM is due to a decrease in the number of “recruitable” cross-bridges that can potentially produce active force. When cross-bridge detachment was accelerated in the presence of 20 mM inorganic phosphate, the Ca^2+^-sensitizing effect of OM (1.0 μM) was markedly decreased in both types of preparations (ΔpCa_50_: ∼0.09 and ∼0.03 pCa units in PLV and PLA, respectively). The present findings suggest that the positive inotropy of OM is more markedly exerted in the ventricle than in the atrium, which results from the strongly bound cross-bridge-dependent allosteric activation of thin filaments.

## Introduction

Heart failure (HF) is a syndrome characterized by symptoms or signs caused by structural or functional abnormalities of the heart, which results in reduced cardiac output (Metra and Teerlink, 2017). The complex clinical syndrome of HF with reduced ejection fraction (HFrEF) requires specifically established medications, such as angiotensin receptor-neprilysin inhibitors, angiotensin-converting enzyme inhibitors, angiotensin receptor blockers, β-blockers, loop diuretics, aldosterone antagonists, hydralazine/isosorbide dinitrate, ivabradine [I_f_ (funny current) channel blocker], and sodium-glucose cotransporter 2 inhibitors (Maddox et al., 2021; McDonagh et al., 2021). In contrast, traditional positive inotropic drugs are known to cause untoward effects (Teerlink et al., 2009); these drugs increase the intracellular Ca^2+^ concentration in myocardial cells, and can cause arrhythmias (Francis et al., 2014).

Omecamtiv mecarbil (OM) was developed as a first-in-class “myosin activator” for the treatment of heart failure in patients with HFrEF (Malik and Morgan, 2011). Several lines of evidence indicate that OM is effective in the treatment of HF by improving cardiac function (Shen et al., 2010; Malik et al., 2011; Teerlink et al., 2016). A recent study in heart failure patients showed that those that were treated with OM had a lower incidence of a composite event of HF hospitalization or death than in those who received placebo (Teerlink et al., 2021). It was originally considered that OM increases cardiac contractility by prolonging the duration of ejection with no influence on the intracellular Ca^2+^ concentration, and that the augmented contractility is the result of the increased transition rate of the OM-bound myosin into the strongly bound, force-generating state (Malik et al., 2011; Nagy et al., 2015). Consistent with this view, Mamidi et al. (2015) proposed that OM lowers the cross-bridge detachment rate, and the resultant strongly bound cross-bridges proceed to activate thin filaments (i.e., thin filament “*on-off*” equilibrium shifting from the “*off*” state towards the “*on*” state; cf. Kobirumaki-Shimozawa et al., 2014). It has likewise been shown that OM increases the release rate of inorganic phosphate (Pi) during the actomyosin ATPase cycle (Liu et al., 2015), promoting the formation of strongly bound cross-bridges, with no change in the ADP dissociation rate (Winkelmann et al., 2015). Subsequently, Rohde et al. (2017) provided evidence that OM stabilizes the pre-powerstroke of myosin; therefore, the compound suppresses myosin’s working stroke and prolongs the time of myosin binding to actin (see Woody et al., 2018 for details). Taken together, these findings support the above interpretation that the compound’s inotropic effect is primarily coupled with cooperative thin filament activation as a result of OM-bound cross-bridges. It can therefore be summarized that OM primarily exerts its positive inotropy as follows: it binds to myosin, increasing the number of strongly bound cross-bridges, and these cross-bridges cooperatively activate thin filaments, thereby recruiting OM-free “recruitable” cross-bridges that can potentially generate active force upon attachment to actin (Lindqvist et al., 2019; Governali et al., 2020; Snoberger et al., 2021) (see Terui et al., 2008, Terui et al., 2010; Kobirumaki-Shimozawa et al., 2014 for “recruitable” cross-bridges).

In porcine hearts, ventricular muscle contains ∼20% α- and ∼80% β-myosin, and atrial muscle ∼90% α- and ∼10% β-myosin (Shchepkin et al., 2020). Shchepkin et al. (2020) likewise showed that OM prolongs the actomyosin attachment duration in ventricular and atrial myosin, with a higher sensitivity in ventricular myosin. However, it has not been investigated whether the cardiotonic effect of OM is greater in the ventricle than in the atrium. In the present study, we investigated the effects of OM in the clinically relevant concentration range (i.e., 0.5 and 1.0 μM; see Teerlink et al., 2011, 2016) on steady-state contractile properties in skinned porcine left ventricular (PLV) and atrial (PLA) muscles. We found that OM increased Ca^2+^ sensitivity by a greater magnitude in PLV than in PLA, and that the Ca^2+^-sensitizing effect of OM was less pronounced under enhanced thin filament cooperative activation. Mechanistic implications are discussed based on the thin filament “*on-off*” regulation and the recently proposed thick filament regulation. Likewise, we discuss the clinical relevance of the present findings focusing on the possible effects of OM on HFrEF.

## Materials and methods

All muscle mechanical experiments were performed at The Jikei University School of Medicine in accordance with the guidelines outlined by the university's Institutional Animal Care and Use Committee. Troponin (Tn) extraction was performed at Hokkaido University. All experiments performed in the present study conform to the Guidelines for Proper Conduct of Animal Experiments of the Science Council of Japan (2006).

### Preparations of skinned muscles

Skinned porcine ventricular (PLV) and atrial (PLA) muscles were prepared based on previously described procedures (Terui et al., 2008, 2010; Matsuba et al., 2009). In brief, porcine hearts (animals, ∼1.0 years) were obtained at a local slaughterhouse. Muscle strips (1–2 mm in diameter and ∼10 mm in length) were dissected from the left ventricle and atrium (as performed on bovine hearts; see Fukuda et al., 2003; 2005a), and were skinned in relaxing solution [5 mM MgATP, 40 mM BES, 1 mM Mg^2+^, 10 mM EGTA, 1 mM dithiothreitol (DTT), 15 mM phosphocreatine (CP), 15 U/ml creatine phosphokinase (CPK), and 180 mM ionic strength (adjusted by K-propionate), pH 7.0, containing 1% (wt/vol) Triton X-100 and 10 mM 2,3-butanedione 2-monoxime (BDM)] overnight at ∼3°C (Terui et al., 2008, 2010; Matsuba et al., 2009). Muscles were stored for up to 3 weeks at −20°C in relaxing solution containing 50% (vol/vol) glycerol. All solutions contained protease inhibitors (0.5 mM PMSF, 0.04 mM leupeptin and 0.01 mM E64) to avoid protein degradation (as in Fukuda et al., 2003, 2005a,b; Terui et al., 2008, 2010; Matsuba et al., 2009).

### Skinned muscle mechanics: Force-pCa protocol

Isometric force was measured using our previously described procedure with PLV (Terui et al., 2008, 2010; Matsuba et al., 2009). Experiments were all conducted at the relatively low temperature of 15°C to minimize rundowns of active force during the time required to perform force-pCa protocols (cf. Fukuda et al., 2003, 2005b). In brief, small thin preparations (∼100 µm in diameter and ∼2 mm in length) were dissected from the PLV and PLA strips for isometric force measurement. Sarcomere length (SL) was measured by laser diffraction during relaxation (as in Fukuda et al., 2003, 2005a,b; Terui et al., 2008, 2010; Matsuba et al., 2009; Inoue et al., 2013), and set at 2.1 µm prior to contraction at each pCa. Passive force was not detected at this SL in either PLV or PLA. pCa was obtained by adjusting the ratio of Ca/EGTA (Fukuda et al., 2003, 2005b; Terui et al., 2008, 2010; Matsuba et al., 2009; Inoue et al., 2013). OM was purchased from ChemScene LLC (Monmouth Junction, NJ, United States). OM was initially dissolved in DMSO, and diluted with individual solutions. The final concentration of DMSO of 0.1% had no effect on Ca^2+^ sensitivity or maximal Ca^2+^-activated force (cf. Fukuda et al., 2000; Terui et al., 2010).

For both PLV and PLA, the muscle preparation was first immersed in high-EGTA (10 mM) relaxing solution. Just before contraction, the preparation was immersed in low-EGTA (0.5 mM) relaxing solution to avoid slowing of contraction and the ensuing damage on sarcomere structures (Terui et al., 2008, 2010; Matsuba et al., 2009; Inoue et al., 2013). The preparation was first activated at pCa 4.5 to obtain maximal Ca^2+^-activated force, followed by relaxation. The preparation was then activated at various pCa’s (from high to low pCa, and lastly at pCa 4.5) to construct the force-pCa curve (i.e., force-pCa protocol). The effect of OM was tested at 0.5 and 1.0 µM, in this order.

Force-pCa curves were fitted to the Hill equation, and the value of the midpoint of the force-pCa curve (i.e., pCa_50_) was used as an index of Ca^2+^ sensitivity (as in Fukuda et al., 2003, 2005b; Terui et al., 2008, 2010; Matsuba et al., 2009; Inoue et al., 2013; Kobirumaki-Shimozawa et al., 2014). We likewise obtained the Hill coefficient (n_H_). Active forces at submaximal Ca^2+^ levels were normalized to maximal force (pCa 4.5) obtained at the beginning or the end of the force-pCa protocol (the rundown in active force during the protocol was less than 10% under all conditions tested). The OM-induced increase in Ca^2+^ sensitivity was quantified by the difference in pCa_50_ obtained in the absence and presence of OM, and expressed as ΔpCa_50_ (in pCa units).

### Measurement of MgADP-induced contraction

Ca^2+^-independent, MgADP-induced active force (ADP-contraction) was measured in PLV and PLA as described previously by us (Fukuda et al., 1998), but at the lower temperature of 15°C. In brief, isometric force was measured in solutions containing 2 mM MgATP, 10 mM MOPS, 2 mM Mg^2+^, 2 mM EGTA, 1 mM DTT, and 150 mM ionic strength (adjusted by K-propionate), pH 7.0. In order to inhibit the rephosphorylation of ADP to ATP by myokinase (Lienhard and Secemski, 1973; Fukuda et al., 1996, 1998), 0.1 mM P^1^,P^5^-di(adenosine-5′)pentaphosphate was added. The maximal concentration of MgADP was set at 20 mM for both PLV and PLA, and submaximal active forces were measured at 0.5, 1, 2 and 3 mM and at 1, 3, 5, 8, 10, and 15 mM for PLV and PLA, respectively. Active forces at submaximal Ca^2+^ levels were normalized to maximal force obtained at the beginning or the end of the force-MgADP protocol (the rundown in active force during the protocol was less than 10% under all conditions tested).

### Treatment with protein kinase A

Protein kinase A (PKA) treatment was performed for PLV and PLA based on our previously published protocol (Fukuda et al., 2005a; Matsuba et al., 2009; Inoue et al., 2013). In brief, after the force-pCa protocol was performed in the absence of OM, the preparation was incubated for 50 min at room temperature in relaxing solution containing purified PKA (catalytic subunit from bovine heart; Sigma-Aldrich Co., St. Louis, MO, United States) at a concentration of 1 U/µl. We previously reported that this protocol effectively phosphorylates TnI and myosin-binding protein C (MyBP-C) in skinned myocardial preparations (Inoue et al., 2013). Then, the force-pCa protocol was repeated two times, in the absence and presence of 1.0 µM OM in this order.

### Treatment with the Tn complex from rabbit fast skeletal muscle

Tn exchange was performed for PLV and PLA based on our previously published procedure (Terui et al., 2008, 2010; Matsuba et al., 2009; Inoue et al., 2013). In brief, the fast skeletal Tn complex (sTn) was extracted from rabbit fast skeletal muscles (based on Ebashi et al., 1971; Eisenberg and Kielley, 1974). sTn extraction was performed at Hokkaido University, and approved by the Animal Care and Use Committee of Hokkaido University (#09-0134). sTn was transported to The Jikei University School of Medicine by air, and stored at −80°C before use. After the force-pCa protocol was performed in the absence of OM, the preparation was bathed in rigor solution (10 mM BES, 150 mM K-propionate, 2.5 mM EGTA, and 5 mM MgCl_2_, pH 7.0) containing 2 mg/ml sTn and 80 mM BDM for 60 min at 25°C. Then, the preparation was washed with normal relaxing solution at 15°C for 10 min with gentle agitation to remove excess sTn. We reported that Tn subunits are almost completely replaced by those from rabbit fast skeletal muscle in skinned cardiac preparations with a diameter of ∼100 µm (Terui et al., 2008; Matsuba et al., 2009; Inoue et al., 2013). The force-pCa protocol was repeated two times, in the absence and presence of 1.0 µM OM in this order.

### Force-pCa protocol in the presence of MgADP or inorganic phosphate

In both PLV and PLA, after the force-pCa protocol was performed, the same procedure in the presence of 3 mM MgADP or 20 mM Pi was repeated (see Terui et al., 2010 for details). Subsequently, the force-pCa protocol was performed with 1.0 µM OM in the presence of 3 mM MgADP or 20 mM Pi. Only when MgADP was present, 0.1 mM P^1^,P^5^-di(adenosine-5′)pentaphosphate was added to both activating and relaxing solutions, with no CP-CPK to maintain the ADP/ATP ratio (see above).

### Statistics

Significant difference was assigned using paired or unpaired Student’s *t* test, or Tukey-Kramer test, as appropriate. As part of the statistical processing, we used EZR (Kanda, 2013), which is a graphical user interface for R (version 4.1.2: The R Foundation for Statistical Computing, Vienna, Austria). Data were expressed as means ± SEM, with *n* representing the number of muscles. Statistical significance was assumed to be *p* < 0.05, *p* < 0.01 and *p* < 0.001. “n.s.” indicates *p* > 0.05 (not significant).

## Results

### Effects of OM on Ca^2+^ sensitivity in PLV and PLA

**FIGURE 1:**
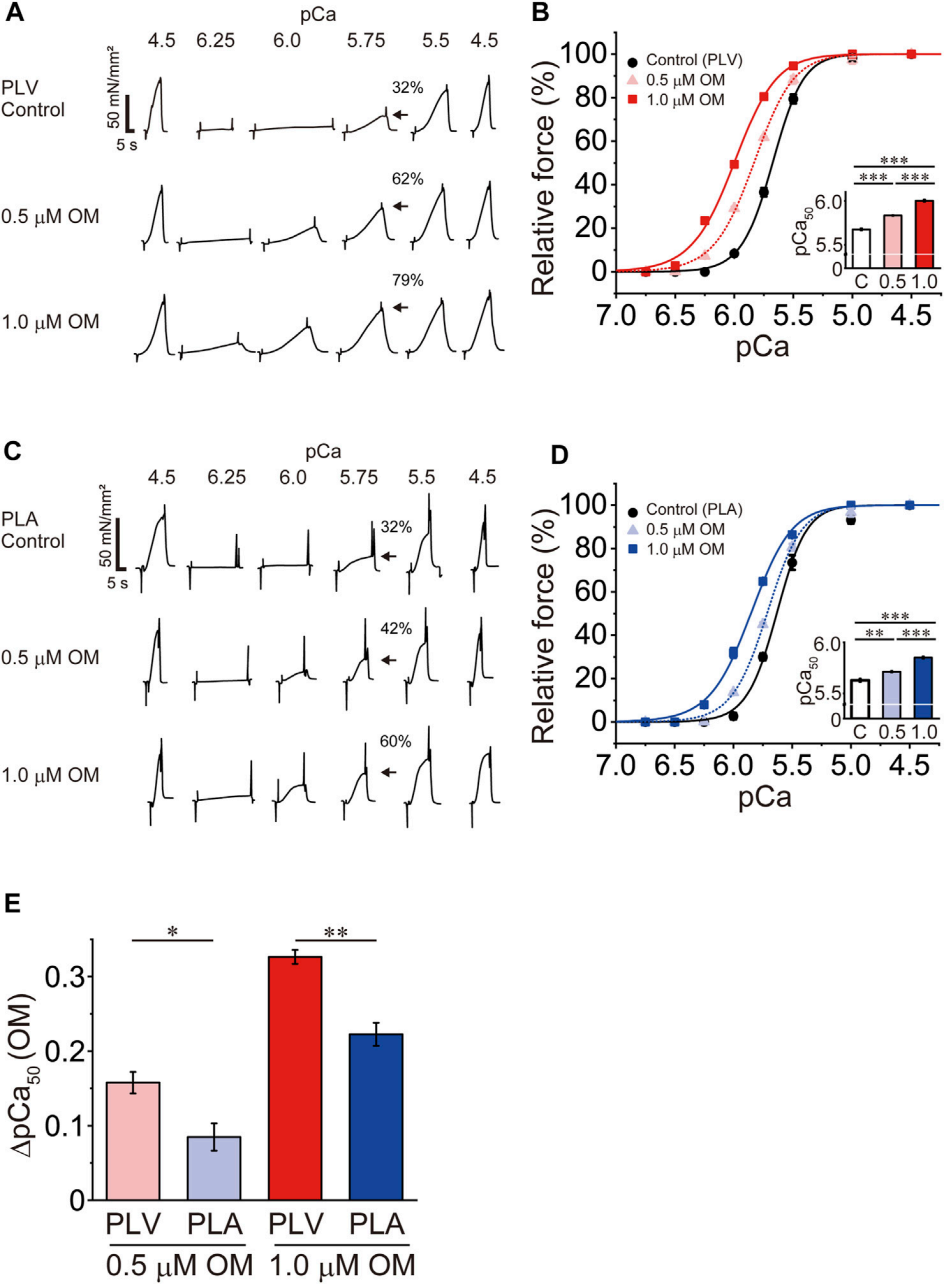
Effects of OM on Ca^2+^ sensitivity in PLV and PLA. **(A)** Typical chart recording showing force-pCa protocols in PLV in the absence and presence of OM at 0.5 and 1.0 μM (in this order). Arrows indicate the percentage of active force at pCa 5.75 compared with the maximum obtained at the end of experiment (pCa 4.5). Note that OM increased submaximal force (i.e., at pCa 6.25, 6.0, 5.75 and 5.5). SL was set at 2.1 μm. Control, without OM. **(B)** Force-pCa curves showing the effects of OM at 0.5 and 1.0 μM on Ca^2+^ sensitivity in PLV. Black circles and solid lines, control (without OM); light red triangles and dotted lines, 0.5 μM OM; red squares and solid lines, 1.0 μM OM. Inset, pCa_50_ values in the absence and presence of OM. C, control (without OM); 0.5, 0.5 μM OM; 1.0, 1.0 μM OM. ***p < 0.001 (Tukey-Kramer test). *n* = 6 for all groups. **(C)** Same as in **(A)** for PLA in the absence and presence of OM at 0.5 and 1.0 μM (in this order). Arrows indicate the percentage at pCa 5.75 compared with the maximum obtained at the end of experiment (pCa 4.5). Note that OM increased submaximal force (i.e., at pCa 6.25, 6.0, 5.75 and 5.5) [as in PLV; see **(A)**]. SL was set at 2.1 μm. Control, without OM. **(D)** Same as in **(B)** for PLA showing the effects of OM at 0.5 and 1.0 μM on Ca^2+^ sensitivity. Black circles and solid lines, control (without OM); light blue triangles and dotted lines, 0.5 μM OM; blue squares and solid lines, 1.0 μM OM. Inset, pCa_50_ values in the absence and presence of OM. C, control (without OM); 0.5, 0.5 μM OM; 1.0, 1.0 μM OM. **p < 0.01; ***p < 0.001 (Tukey-Kramer test). *n* = 5 for all groups. **(E)** Graph comparing the Ca^2+^-sensitizing effect of OM (ΔpCa_50_) between PLV and PLA at 0.5 and 1.0 μM. At both concentrations, ΔpCa_50_ was greater for PLV than PLA. *p < 0.05; **p < 0.01 (unpaired Student’s *t* test).

First, we investigated the effects of OM on Ca^2+^ sensitivity in PLV and PLA under the condition with no perturbation (SL 2.1 μm; see **Materials and Methods**). We tested these effects in a paired manner, i.e., one skinned muscle preparation, either PLV or PLA, at no OM, and then at 0.5 and 1.0 μM in this order. In PLV, under the control condition without OM, isometric force started to rise at pCa 6.0 (8%), and became 32% and 76% at pCa 5.75 and 5.5, respectively (Figure 1A: percentages compared to maximal force). OM increased submaximal forces; i.e., the force levels were 28% and 62%, respectively, at pCa 6.0 and 5.75 in the presence of 0.5 μM OM, and 49% and 79%, respectively, in the presence of 1.0 μM OM. Figure 1B shows force-pCa curves in the absence and presence of OM (Table 1). The mid-point of the force-pCa curve (pCa_50_) was 5.67 ± 0.01 in the absence of OM, which was slightly greater than that obtained in our previous reports using PLV at SL 1.9 μm (Terui et al., 2008, 2010; Matsuba et al., 2009); presumably this was coupled with the SL-dependent increase in Ca^2+^ sensitivity (see Kobirumaki-Shimozawa et al., 2014 and references therein). OM increased Ca^2+^ sensitivity in a concentration-dependent manner, left shifting pCa_50_ by 0.16 ± 0.01 and 0.33 ± 0.01 pCa units (ΔpCa_50_) at 0.5 and 1.0 μM (*p* < 0.001), respectively (Table 1). Maximal force was not significantly affected by either concentration of OM in PLV (Table 1).

**TABLE 1:** Summary of the effects of OM on maximal force, Ca^2+^ sensitivity and n_H_ in PLV and PLA.

	Maximal force (mN/mm^2^)	pCa_50_	ΔpCa_50_	n_H_	*n*
PLV	62.4 ± 1.9	5.67 ± 0.01		3.33 ± 0.12	6
PLV (0.5 μM OM)	66.4 ± 1.7	5.83 ± 0.003***	0.16 ± 0.01	2.53 ± 0.09	6
PLV (1.0 μM OM)	70.1 ± 2.7	6.00 ± 0.01***, ^###^	0.33 ± 0.01^###^	2.42 ± 0.07	6
PLA	40.9 ± 0.9^†††^	5.63 ± 0.02		3.38 ± 0.22	5
PLA (0.5 μM OM)	48.1 ± 1.6*	5.71 ± 0.01**	0.08 ± 0.02^†^	2.90 ± 0.13	5
PLA (1.0 μM OM)	48.9 ± 1.9*	5.85 ± 0.01***, ^###^	0.22 ± 0.02^###, ††^	2.50 ± 0.11	5

Maximal force was obtained by activating muscle at pCa 4.5 prior to or at the end of construction of the force-pCa curve. ΔpCa_50_, shift of pCa_50_ upon application of OM. *n*, number of experiments. The effects of OM were tested at 0.5 and 1.0 μM, in this order. *, vs. control (without OM) (Tukey-Kramer test); ^#^, vs. 0.5 μM OM (pCa_50_, Tukey-Kramer test; ΔpCa_50_, paired Student’s *t* test); ^†^, vs. PLV (unpaired Student’s *t* test). Single, double and triple symbols denote significant difference at *p* < 0.05, 0.01 and 0.001, respectively.

In PLA, under the control condition without OM, active force increased in a manner similar to that in PLV, i.e., 32% and 74% at pCa 5.75 and 5.5, respectively (Figure 1C: percentages compared to maximal force). OM increased submaximal forces; i.e., the force levels were 16% and 42%, respectively, at pCa 6.0 and 5.75 in the presence of 0.5 μM OM, and 37% and 60%, respectively, in the presence of 1.0 μM OM. Figure 1D shows force-pCa curves in the absence and presence of OM (Table 1). OM increased Ca^2+^ sensitivity by left shifting pCa_50_ in a concentration-dependent manner; however, the magnitude was less than that in PLV, i.e., ΔpCa_50_ 0.08 ± 0.02 and 0.22 ± 0.02 pCa units at 0.5 and 1.0 μM OM (*p* < 0.001), respectively (Table 1). Unlike in PLV, OM significantly increased maximal force in PLA by ∼20% at both concentrations (*p* < 0.05; Table 1).

Figure 1E compares the ΔpCa_50_ values in PLV vs. PLA at 0.5 and 1.0 μM. The compound’s Ca^2+^-sensitizing effect was significantly more pronounced in PLV than in PLA at both concentrations. It can therefore be said that OM increases Ca^2+^ sensitivity in a concentration-dependent manner in both PLV and PLA, with the effect more pronounced in PLV.

### Effects of OM on Ca^2+^ sensitivity in PLV and PLA following PKA treatment

**FIGURE 2:**
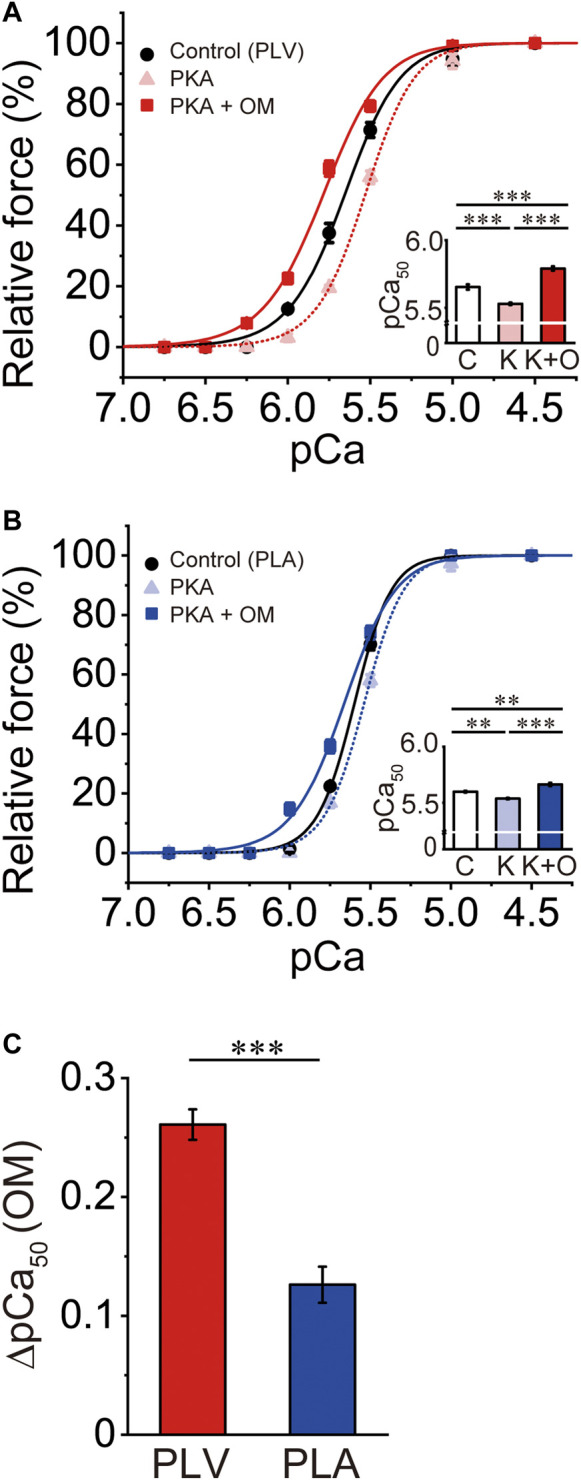
Effects of OM on Ca^2+^ sensitivity in PLV and PLA following PKA treatment. **(A)** Force-pCa curves showing the effect of 1.0 μM OM on Ca^2+^ sensitivity following PKA treatment in PLV. Black circles and solid lines, control (before PKA treatment); light red triangles and dotted lines, after PKA treatment; red squares and solid lines, 1.0 μM OM after PKA treatment. Inset, pCa_50_ values under varying conditions. C, control (without OM); K, PKA treatment; K + O, 1.0 μM OM after PKA treatment. ***p < 0.001 (Tukey-Kramer test). *n* = 6 for all groups. **(B)** Same as in **(A)** for PLA showing the effect of 1.0 μM OM on Ca^2+^ sensitivity following PKA treatment. Black circles and solid lines, control (before PKA treatment); light blue triangles and dotted lines, after PKA treatment; blue squares and solid lines, 1.0 μM OM after PKA treatment. Inset, pCa_50_ values under varying conditions. C, control (without OM); K, PKA treatment; K + O, 1.0 μM OM after PKA treatment. **p < 0.01; ***p < 0.001 (Tukey-Kramer test). *n* = 5 for all groups. **(C)** Graph comparing the Ca^2+^-sensitizing effect of 1.0 μM OM (ΔpCa_50_) following PKA treatment between PLV and PLA. ***p < 0.001 (unpaired Student’s *t* test).

PKA treatment decreases Ca^2+^ sensitivity in cardiac muscle, due to phosphorylation of TnI and the ensuing reduction of the TnC-TnI interaction (see Solaro and Rarick, 1998; Matsuba et al., 2009; Inoue et al., 2013 and references therein). It has likewise been reported that PKA phosphorylates MyBP-C and accelerates cross-bridge cycling (Jeacocke and England, 1980; Harris et al., 2002; Tong et al., 2008). Considering that the mammalian heart is constantly under the influence of β-adrenergic stimulation, we next investigated whether or not the Ca^2+^-sensitizing effect of OM is altered in PLV and PLA following PKA treatment. Consistent with our previous study (Matsuba et al., 2009), PKA decreased Ca^2+^ sensitivity in PLV by right shifting pCa_50_ by 0.12 ± 0.02 pCa units (Figure 2A; Table 2). OM at 1.0 μM increased Ca^2+^ sensitivity following PKA treatment by 0.26 ± 0.01 pCa units. The magnitude of increase in Ca^2+^ sensitivity was ∼20% (*p* < 0.01) less than that obtained with no PKA treatment (Tables 1, 2). In PLA, PKA decreased Ca^2+^ sensitivity by right shifting pCa_50_ by 0.06 ± 0.01 pCa units, i.e., ∼50% less compared with PLV (Figure 2B; Table 2). As found in PLV, OM at 1.0 μM increased Ca^2+^ sensitivity following PKA treatment by 0.13 ± 0.02 pCa units, i.e., ∼43% (*p* < 0.01) less than that obtained with no PKA treatment (Tables 1, 2). Following PKA treatment, therefore, OM increases Ca^2+^ sensitivity in both PLV and PLA, with a greater increase in PLV (Figure 2C); compared with no PKA treatment, however, the effect is less in both preparations. OM did not significantly alter maximal force in PLV or PLA following PKA treatment (Table 2).

**TABLE 2:** Summary of the effects of OM on maximal force, Ca^2+^ sensitivity and n_H_ in PLV and PLA following PKA treatment.

	Maximal force (mN/mm^2^)	pCa_50_	ΔpCa_50_	n_H_	*n*
PLV	60.7 ± 2.6	5.65 ± 0.02		2.61 ± 0.12	6
PLV (PKA)	75.8 ± 5.2	5.53 ± 0.01***	−0.12 ± 0.02	2.86 ± 0.15	6
PLV (PKA + 1.0 μM OM)	90.4 ± 4.0**	5.79 ± 0.02***, ^###^	0.26 ± 0.01^♭♭^	2.47 ± 0.12	6
PLA	40.8 ± 0.9	5.60 ± 0.01		3.76 ± 0.11	5
PLA (PKA)	52.1 ± 3.1*	5.54 ± 0.01**	−0.06 ± 0.01^†^	3.45 ± 0.26	5
PLA (PKA + 1.0 μM OM)	53.2 ± 3.0*	5.66 ± 0.01**, ^###^	0.13 ± 0.02^†††, ♭♭^	2.70 ± 0.12	5

Maximal force was obtained by activating muscle at pCa 4.5 prior to or at the end of construction of the force-pCa curve. ΔpCa_50_, shift of pCa_50_ upon PKA treatment (PKA) or 1.0 μM OM application following PKA treatment. *n*, number of experiments. *, vs. control (without OM) (Tukey-Kramer test); ^#^, vs. PKA treatment without OM (Tukey-Kramer test); ^†^, vs. PLV (unpaired Student’s *t* test); ^♭^, vs. 1.0 μM OM in Table 1 (unpaired Student’s *t* test). Single, double and triple symbols denote significant difference at *p* < 0.05, 0.01 and 0.001, respectively.

### Effects of OM on ADP-contraction in PLV and PLA

We previously reported that increasing the MgADP concentration generates active force in the absence of Ca^2+^ (+ATP and +EGTA); this is a phenomenon known as ADP-induced contraction or “ADP-contraction” which occurs via thin filament cooperative activation by the strongly bound, actomyosin-ADP complex (see e.g., Shimizu et al., 1992; Fukuda et al., 1996, 1998). To confirm whether or not OM increases active force by modulating the processes downstream of Ca^2+^-binding to TnC in the cross-bridge cycle, we investigated the effects of OM on ADP-contraction in PLV and PLA.

**FIGURE 3:**
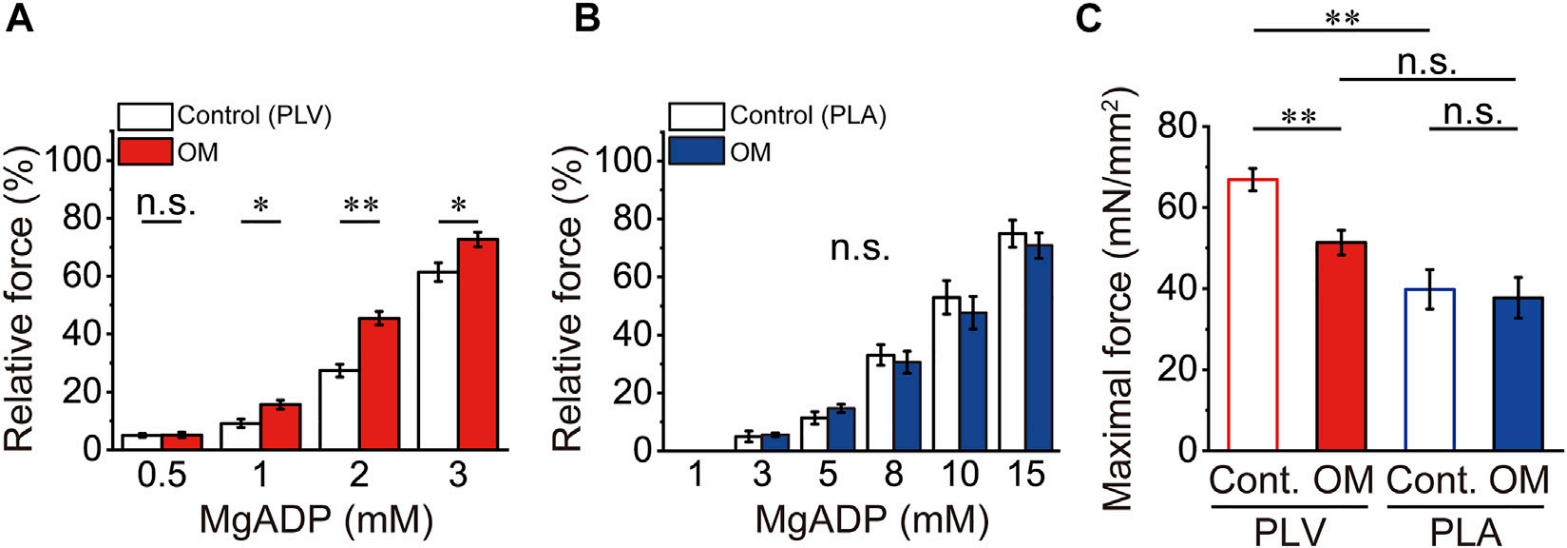
Effects of OM on ADP-contraction in PLV and PLA. **(A)** Graph showing the effect of 1.0 μM OM on Ca^2+^-independent active force in PLV upon increasing the MgADP concentration at 2 mM MgATP. Note that submaximal forces at 1, 2, and 3 mM MgADP were increased in the presence of 1.0 μM OM. *p < 0.05; **p < 0.01 (unpaired Student’s *t* test). Submaximal forces were normalized compared with that at 20 mM MgADP with or without OM [see **(C)**]. White and red, in the absence (Control) and presence of OM. *n* = 5 for both groups. **(B)** Same as in **(A)** for PLA. Unlike in PLV, submaximal forces were unaffected by 1.0 μM OM. Submaximal forces were normalized compared with that at 20 mM MgADP with or without OM [see **(C)**]. White and blue, in the absence (Control) and presence of OM. *n* = 5 for both groups. **(C)** Comparison of maximal force values at 20 mM MgADP with and without 1.0 μM OM in PLV (red) and PLA (blue). Maximal force was obtained by activating muscle at 20 mM MgADP prior to investigation of submaximal forces. **p < 0.01 (unpaired Student’s *t* test).

In PLV, ADP-induced active force increased in a concentration-dependent manner, i.e., 9.1% ± 1.5%, 27.4% ± 2.2% and 61.4% ± 3.2% at 1, 2 and 3 mM MgADP (compared to the maximum at 20 mM MgADP), respectively, in the presence of 2 mM MgATP (Figure 3A). This is similar to our earlier finding using bovine left ventricular muscle (Fukuda et al., 1998), but slightly less sensitive to MgADP, due presumably to a decrease in the apparent binding affinity for MgADP at the lower temperature of 15°C in the present study compared to 25°C in Fukuda et al. (1998). OM at 1.0 μM significantly augmented submaximal forces of ADP-contraction, resulting in 15.6% ± 1.5%, 45.4% ± 2.3% and 72.7% ± 2.5% at 1, 2 and 3 mM MgADP. In PLA, MgADP sensitivity was markedly less than that in PLV; submaximal forces were 11.4% ± 2.1%, 33.1% ± 3.6%, 53.0% ± 5.8% and 74.9% ± 4.6% at 5, 8, 10, and 15 mM MgADP, respectively (compared to the maximum at 20 mM MgADP) (Figure 3B). Unlike in PLV, submaximal forces were not significantly affected by 1.0 μM OM at all MgADP concentrations tested. Therefore, OM enhanced Ca^2+^-independent, strongly bound cross-bridge-dependent contraction in PLV, but not in PLA. It should likewise be noted that OM significantly decreased maximal force of ADP-contraction at 20 mM in PLV (by ∼23%), but not in PLA (Figure 3C).

### Effects of OM on Ca^2+^ sensitivity in PLV and PLA under enhanced activation condition

**FIGURE 4:**
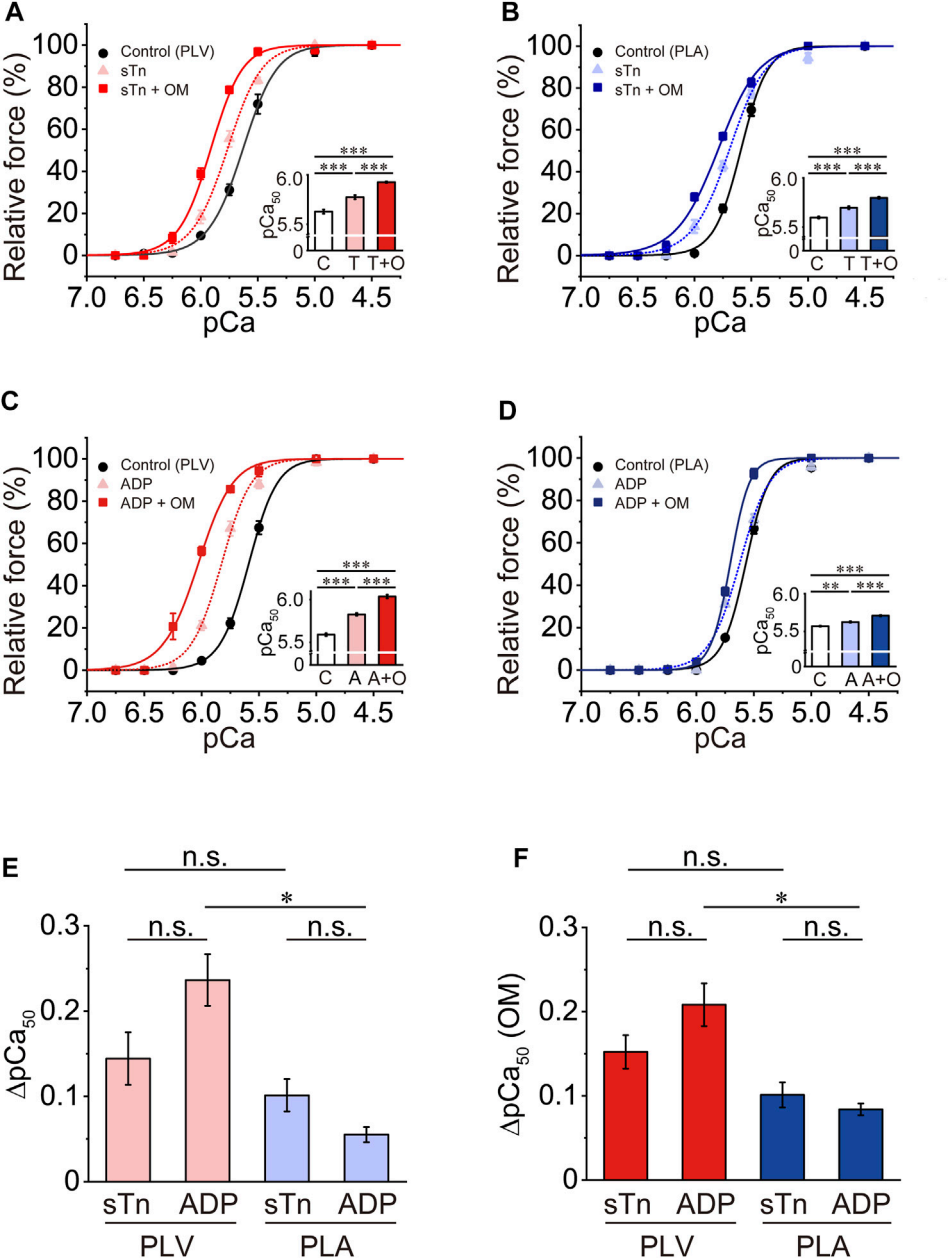
Effects of OM on Ca^2+^ sensitivity in PLV and PLA under enhanced activation condition. **(A)** Force-pCa curves showing the effect of 1.0 μM OM on Ca^2+^ sensitivity in PLV following sTn treatment. Black circles and solid lines, control (before sTn treatment); light red triangles and dotted lines, after sTn treatment; red squares and solid lines, 1.0 μM OM after sTn treatment. Inset, pCa_50_ values indicating Ca^2+^ sensitivity under varying conditions. C, control (without OM); T, sTn treatment; T + O, 1.0 μM OM after sTn treatment. ***p < 0.001 (Tukey-Kramer test). *n* = 6 for all groups. **(B)** Same as in **(A)** for PLA showing the effect of 1.0 μM OM on Ca^2+^ sensitivity following sTn treatment. Black circles and solid lines, control (before sTn treatment); light blue triangles and dotted lines, after sTn treatment; blue squares and solid lines, 1.0 μM OM after sTn treatment. Inset, pCa_50_ values under varying conditions. C, control (without OM); T, sTn treatment; T + O, 1.0 μM OM after sTn treatment. ***p < 0.001 (Tukey-Kramer test). *n* = 5 for all groups. **(C)** Force-pCa curves showing the effect of 1.0 μM OM on Ca^2+^ sensitivity in PLV following 3 mM MgADP application. Black circles and solid lines, control (before MgADP application); light red triangles and dotted lines, after MgADP application; red squares and solid lines, 1.0 μM OM after MgADP application. Inset, pCa_50_ values under varying conditions. C, control (without OM); A, MgADP application; A + O, 1.0 μM OM after MgADP application. ***p < 0.001 (Tukey-Kramer test). *n* = 4 for all groups. **(D)** Same as in **(A)** for PLA showing the effect of 1.0 μM OM on Ca^2+^ sensitivity following 3 mM MgADP application. Black circles and solid lines, control (before MgADP application); light blue triangles and dotted lines, after MgADP application; blue squares and solid lines, 1.0 μM OM after MgADP application. Inset, pCa_50_ under varying conditions. C, control (without OM); A, MgADP application; A + O, 1.0 μM OM after MgADP application. **p < 0.01; ***p < 0.001 (Tukey-Kramer test). *n* = 5 for all groups. **(E)** Graph showing the increased Ca^2+^ sensitivity (ΔpCa_50_) following sTn treatment (sTn) vs. MgADP application (ADP) in PLV and PLA. *p < 0.05 (unpaired Student’s t test). Data obtained from **(A**–**D)**. **(F)** Graph showing the Ca^2+^-sensitizing effect of 1.0 μM OM [ΔpCa_50_ (OM)] following sTn treatment (sTn) vs. MgADP application (ADP) in PLV and PLA. *p < 0.05 (unpaired Student’s *t* test). Data obtained from **(A–D)**.

We previously reported that sTn treatment enhances thin filament cooperative activation and accordingly increases Ca^2+^ sensitivity in PLV (Terui et al., 2008, 2010). We then investigated the Ca^2+^-sensitizing effect of OM under a condition where thin filament cooperative activation is enhanced following sTn treatment. Consistent with our previous results (Terui et al., 2008, 2010), sTn treatment increased Ca^2+^ sensitivity in PLV by left shifting pCa_50_ by 0.14 ± 0.03 pCa units (Figure 4A; Table 3). OM at 1.0 μM further increased Ca^2+^ sensitivity, but ΔpCa_50_ was only 0.16 ± 0.02 pCa units (*p* < 0.001 compared with the value under the control condition; i.e., 0.33 ± 0.01 pCa units in Figure 1B). In PLA, sTn treatment increased Ca^2+^ sensitivity by left shifting pCa_50_ by 0.10 ± 0.02 pCa units (Figure 4B; Table 3). As in the PLV finding, ΔpCa_50_ was less following sTn reconstitution with a value of 0.10 ± 0.01 pCa units when compared with that under the control condition (0.22 ± 0.02 pCa units in Figure 1D; *p* < 0.001).

**TABLE 3:** Summary of the effects of OM on maximal force, Ca^2+^ sensitivity and n_H_ in PLV and PLA under enhanced activation condition.

	Maximal force (mN/mm^2^)	pCa_50_	ΔpCa_50_	n_H_	*n*
PLV	60.9 ± 1.5	5.63 ± 0.02		3.10 ± 0.32	6
PLV (sTn)	70.5 ± 3.9	5.77 ± 0.02***	0.14 ± 0.03	3.01 ± 0.19	6
PLV (sTn + 1.0 μM OM)	77.3 ± 6.1	5.93 ± 0.01***, ^###^	0.16 ± 0.02^♭♭♭^	3.37 ± 0.30	6
PLA	44.1 ± 0.9	5.60 ± 0.01		3.77 ± 0.28	5
PLA (sTn)	57.8 ± 3.8*	5.70 ± 0.02***	0.10 ± 0.02	2.73 ± 0.15	5
PLA (sTn + 1.0 μM OM)	58.0 ± 3.2*	5.80 ± 0.01***, ^###^	0.10 ± 0.01^♭♭♭^	2.43 ± 0.15	5
PLV	62.1 ± 2.0	5.59 ± 0.02		3.49 ± 0.11	4
PLV (ADP)	91.5 ± 5.6*	5.83 ± 0.02***	0.24 ± 0.03	3.39 ± 0.21	4
PLV (ADP + 1.0 μM OM)	94.5 ± 5.6**	6.04 ± 0.02***, ^‡‡‡^	0.21 ± 0.03^♭^	2.89 ± 0.18	4
PLA	41.8 ± 1.6	5.57 ± 0.01		4.23 ± 0.25	5
PLA (ADP)	53.3 ± 3.6	5.62 ± 0.01**	0.06 ± 0.01^†^	3.36 ± 0.20	5
PLA (ADP + 1.0 μM OM)	61.2 ± 2.7**	5.71 ± 0.01***, ^‡‡‡^	0.08 ± 0.01^†, ♭♭♭^	6.10 ± 1.10	5

Maximal force was obtained by activating muscle at pCa 4.5 prior to or at the end of construction of the force-pCa curve. ΔpCa_50_, shift of pCa_50_ upon sTn treatment (sTn), 3 mM MgADP application (ADP) or 1.0 μM OM application after sTn treatment or MgADP application. *n*, number of experiments. *, vs. control (no sTn treatment or MgADP application) (Tukey-Kramer test); ^#^, vs. sTn treatment (without OM) (Tukey-Kramer test); ^‡^, vs. MgADP application (without OM) (Tukey-Kramer test); ^†^, vs. PLV (unpaired Student’s *t* test); ^♭^, vs. 1.0 μM OM in Table 1 (unpaired Student’s *t* test). Single, double and triple symbols denote significant difference at *p* < 0.05, 0.01 and 0.001, respectively.

MgADP is known to augment myocardial contraction by increasing Ca^2+^ sensitivity via formation of strongly bound cross-bridges (actomyosin-ADP complex) and the ensuing enhancement of thin filament cooperative activation (see e.g., Fukuda et al., 1998, 2000; Terui et al., 2010). We then investigated whether or not the Ca^2+^-sensitizing effect of OM is diminished in the presence of MgADP in PLV and PLA, as with sTn treatment. Consistent with our previous results (Terui et al., 2010), 3 mM MgADP increased Ca^2+^ sensitivity in PLV by left shifting pCa_50_ by 0.24 ± 0.03 pCa units (Figure 4C; Table 3). Ca^2+^ sensitivity was further increased by 1.0 μM OM, but ΔpCa_50_ was significantly (*p* < 0.05) less with 0.21 ± 0.03 pCa units when compared with that under the control condition (i.e., 0.33 ± 0.01 pCa units in Figure 1B). In PLA, 3 mM MgADP increased Ca^2+^ sensitivity by left shifting pCa_50_ by 0.06 ± 0.01 pCa units, significantly less than that in PLV (Figure 4D; Table 3). As in the PLV finding, the Ca^2+^-sensitizing effect of OM was significantly (*p* < 0.001) less in the presence of MgADP with ΔpCa_50_ 0.08 ± 0.01 pCa units [compared with the value (0.22 ± 0.02 pCa units) under the control condition; cf. Figure 1D].

The Ca^2+^ sensitization by sTn treatment vs. 3 mM MgADP application in PLV and PLA (expressed in ΔpCa_50_) is compared in Figure 4E. We found that ΔpCa_50_ was statistically insignificant between sTn treatment and MgADP application in PLV and PLA, suggesting that a similar magnitude of thin filament cooperative activation is attained by the two different procedures in both types of preparations (cf. Terui et al., 2010; Kobirumaki-Shimozawa et al., 2014). The magnitude of the OM-induced increase in Ca^2+^ sensitivity (ΔpCa_50_) was likewise insignificant following sTn treatment and in the presence of 3 mM MgADP in both PLV and PLA (Figure 4F). The greater Ca^2+^-sensitizing effect of OM in PLV than in PLA was retained in the presence of 3 mM MgADP; however, the effect between PLV and PLA was insignificant following sTn treatment (Figure 4F). OM did not affect maximal force following sTn treatment or MgADP application in both PLV and PLA (Table 3).

### Effects of OM on Ca^2+^ sensitivity in PLV and PLA under depressed activation condition

**FIGURE 5:**
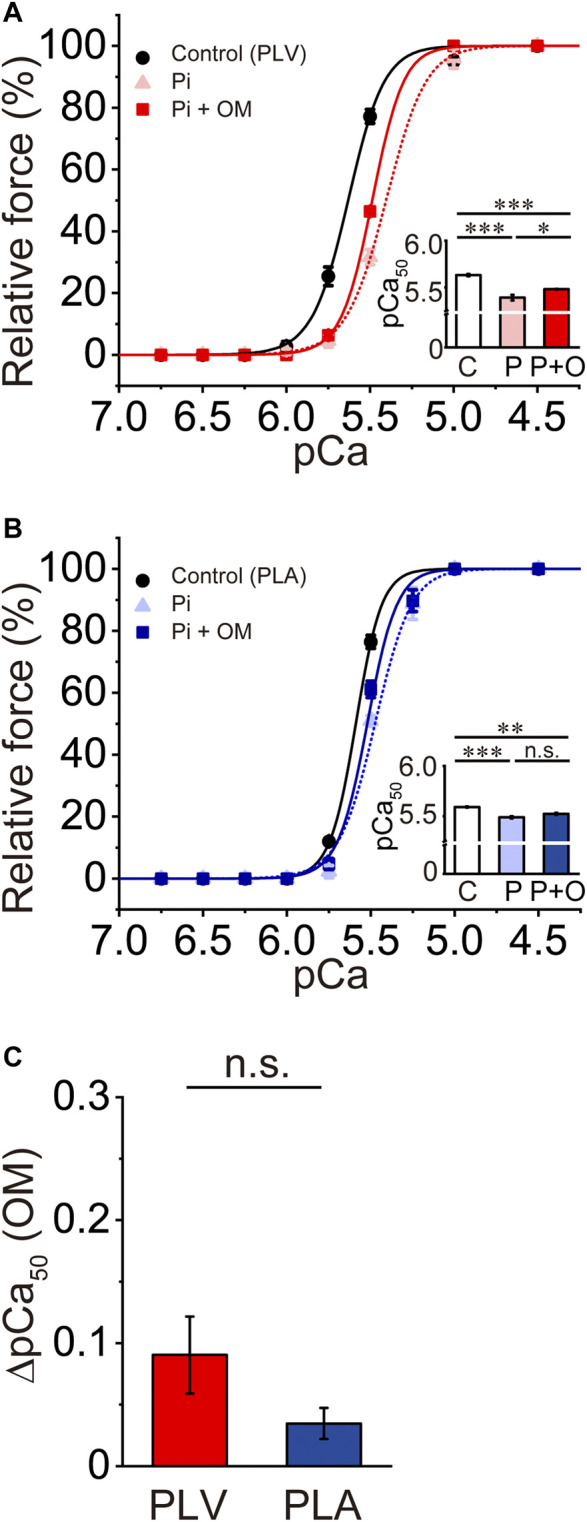
Effects of OM on Ca^2+^ sensitivity in PLV and PLA under depressed activation condition. **(A)** Force-pCa curves showing the effect of 1.0 μM OM on Ca^2+^ sensitivity in PLV following 20 mM Pi application. Black circles and solid lines, control (before Pi application); light red triangles and dotted lines, after Pi application; red squares and solid lines, 1.0 μM OM after Pi application. Inset, pCa_50_ values indicating Ca^2+^ sensitivity under varying conditions. C, control (without OM); P, Pi; P + O, 1.0 μM OM after Pi application. *p < 0.05; ***p < 0.001 (Tukey-Kramer test). *n* = 5 for all groups. **(B)** Same as in **(A)** showing the effect of 1.0 μM OM on Ca^2+^ sensitivity in PLA following 20 mM Pi application. Black circles and solid lines, control (before Pi application); light blue triangles and dotted lines, after Pi application; blue squares and solid lines, 1.0 μM OM after Pi application. Inset, pCa_50_ values indicating Ca^2+^ sensitivity under varying conditions. C, control (without OM); P, Pi; P + O, 1.0 μM OM after Pi application. ***p < 0.001 (Tukey-Kramer test). *n* = 5 for all groups. **(C)** Graph comparing the Ca^2+^-sensitizing effect of 1.0 μM OM (ΔpCa_50_) between PLV and PLA following 20 mM Pi application.

Pi decreases Ca^2+^ sensitivity in cardiac muscle, by accelerating the detachment of bound cross-bridges (see e.g., Fukuda et al., 1996, 1998; Terui et al., 2010). Finally, we investigated whether or not the Ca^2+^-sensitizing effect of OM is altered in PLV and PLA under the depressed condition in the presence of 20 mM Pi. Consistent with our previous results (Terui et al., 2010), 20 mM Pi decreased Ca^2+^ sensitivity in PLV by right shifting pCa_50_ by 0.24 ± 0.04 pCa units (Figure 5A; Table 4). OM at 1.0 μM increased Ca^2+^ sensitivity by 0.09 ± 0.03 pCa units, which was markedly smaller than that obtained under the control condition (i.e., *p* < 0.01 compared with 0.33 ± 0.01 pCa units obtained under the control condition in Figure 1B). In PLA, 20 mM Pi decreased Ca^2+^ sensitivity by right shifting pCa_50_ by 0.10 ± 0.01 pCa units (Figure 5B; Table 4). As in the PLV finding, ΔpCa_50_ in PLA was less in the presence of Pi with merely 0.03 ± 0.01 pCa units (*p* < 0.001 compared with 0.22 ± 0.02 pCa units obtained under the control condition in Figure 1D). The difference between PLV and PLA in the Ca^2+^-sensitizing effect of OM in the presence of Pi was insignificant (*p* > 0.05) (Figure 5C). It should be noted in the presence of Pi that maximal force was significantly augmented by OM in PLA, but not in PLV (Table 4).

**TABLE 4:** Summary of the effects of OM on maximal force, Ca^2+^ sensitivity and n_H_ in PLV and PLA under depressed activation condition.

	Maximal force (mN/mm^2^)	pCa_50_	ΔpCa_50_	n_H_	*n*
PLV	63.8 ± 1.8	5.63 ± 0.01		4.10 ± 0.30	5
PLV (Pi)	40.3 ± 3.2***	5.39 ± 0.03***	-0.24 ± 0.04	3.66 ± 0.32	5
PLV (Pi + 1.0 μM OM)	50.0 ± 2.9*	5.48 ± 0.003***, ^#^	0.09 ± 0.03^♭♭^	4.26 ± 0.28	5
PLA	39.9 ± 0.8	5.59 ± 0.01		5.59 ± 0.35	5
PLA (Pi)	20.8 ± 0.3***	5.49 ± 0.01***	-0.10 ± 0.01^†^	4.22 ± 0.36	5
PLA (Pi + 1.0 μM OM)	27.3 ± 0.8***, ^###^	5.52 ± 0.01**	0.03 ± 0.01^♭♭♭^	5.53 ± 0.96	5

Maximal force was obtained by activating muscle at pCa 4.5 prior to or at the end of construction of the force-pCa curve. ΔpCa_50_, shift of pCa_50_ upon 20 mM Pi application (Pi) or 1.0 μM OM application after Pi application. *n*, number of experiments. *, vs. control (without OM) (Tukey-Kramer test); ^#^, vs. Pi application without OM (Tukey-Kramer test); ^†^, vs. PLV (unpaired Student’s *t* test); ^♭^, vs. 1.0 μM OM in Table 1 (unpaired Student’s *t* test). Single, double and triple symbols denote significant difference at *p* < 0.05, 0.01 and 0.001, respectively.

## Discussion

The findings of the present study are four-fold: 1) OM at clinically relevant concentrations increased submaximal forces in a concentration-dependent manner in PLV and PLA, with a greater magnitude in PLV, 2) OM augmented Ca^2+^-independent, strongly bound cross-bridge-dependent submaximal forces in PLV, but not in PLA, 3) OM’s Ca^2+^-sensitizing effect was attenuated in both PLV and PLA upon enhanced thin filament cooperative activation, directly by sTn treatment and indirectly by MgADP application, and 4) Pi markedly decreased the Ca^2+^-sensitizing effect of OM.

First, OM increased Ca^2+^ sensitivity at 0.5 and 1.0 μM in a concentration-dependent manner in PLV (Figure 1). The magnitude of the increase in Ca^2+^ sensitivity is largely consistent with the findings of previous reports by others using ventricular muscles of various animal species [i.e., rat (Nagy et al., 2015; Kieu et al., 2019), mouse (Mamidi et al., 2015; Utter et al., 2015), guinea pig (Gollapudi et al., 2017) and human (Mamidi et al., 2017)]. Notably, Mamidi et al. (2017) showed that OM at 0.5 and 1.0 μM enhances active force at submaximal Ca^2+^ levels in a concentration-dependent manner in skinned human left ventricular muscle. To the knowledge of the authors, the present study is the first to demonstrate that OM increases Ca^2+^ sensitivity in atrial muscle (PLA) (Figure 1). Considering the previous report by Shchepkin et al. (2020) which demonstrated that OM prolongs the actomyosin attachment duration in ventricular and atrial myosin, with a higher sensitivity in ventricular myosin, the differential Ca^2+^-sensitizing effect of OM in PLV vs. PLA is likely to reflect the varying sensitivity of OM in the prolongation of the actomyosin attachment duration. Given the possible mechanism of action of OM in that it allosterically promotes the formation of force-generating cross-bridges via thin filament cooperative activation (see **Introduction**), the following mechanism can likewise be proposed to account for the differential effect of OM in PLV vs. PLA: namely, the actomyosin attachment duration in the presence of OM is longer for ventricular than atrial myosin in the porcine heart (at 0.5 and 1.0 μM; see Shchepkin et al., 2020), presumably because the attachment duration is intrinsically longer for the former than the latter. It is therefore likely that in PLV, the thin filament “*on-off*” equilibrium is more easily shifted towards the “*on*” state by OM-bound cross-bridges, and, accordingly, a substantial number of force-generating cross-bridges are formed (i.e., causing increased Ca^2+^ sensitivity). This scenario may be supported by the result that maximal force was significantly higher in PLV than in PLA (by ∼1.5-fold; Table 1) (see a similar tendency on ventricular vs. atrial muscles from bovine hearts; Fukuda et al., 2003). And maximal force was increased by ∼20% upon application of OM at 0.5 and 1.0 μM in PLA, but not in PLV (Table 1), reflecting, presumably, the transition of “recruitable” cross-bridges to force-generating cross-bridges via enhanced thin filament cooperative activation. Indeed, it is widely regarded that in both cardiac and skeletal muscles, Ca^2+^ alone cannot fully activate thin filaments; it is the strongly bound cross-bridges, such as rigor cross-bridges (e.g., Bremel and Weber, 1972), *N*-ethylmaleimide-treated myosin subfragment 1 (e.g., Nagashima and Asakura, 1982) and the actomyosin-ADP complex (e.g., Cooke and Pate, 1985; Shimizu et al., 1992; Fukuda et al., 1998), that augment maximal force, cooperatively with Ca^2+^.

In mammals, the heart is consistently under the influence of β-adrenergic stimulation *in vivo*. As discussed in our earlier papers (e.g., Matsuba et al., 2009; Kobirumaki-Shimozawa et al., 2014), a decrease in the Ca^2+^-binding affinity to TnC following PKA-dependent phosphorylation of TnI results from the reduced TnC-TnI interaction. We found that OM increased Ca^2+^ sensitivity in both PLV and PLA following PKA treatment, but the effect was significantly less pronounced in both preparations compared with no PKA treatment (by ∼20% and ∼43% in PLV and PLA, respectively; cf. Figure 1) (Figure 2). Because OM directly binds to myosin (e.g., Winkelmann et al., 2015), it is unlikely that the attenuation of the effect of OM is coupled with a decrease in the Ca^2+^-binding affinity of TnC. It is important that PKA phosphorylates MyBP-C (e.g., Jeacocke and England, 1980) and titin (e.g., Yamasaki et al., 2002; Fukuda et al., 2005a), other than TnI; phosphorylation of MyBP-C may loosen its constraint on the thick filament backbone and allow myosin heads to interact with thin filaments (Colson et al., 2008; Kensler et al., 2017; McNamara et al., 2019). Therefore, OM’s effect may be blunted in both PLV and PLA following PKA treatment because fewer myosin heads would be recruited to thin filaments due to a change in the thick filament-based regulation (see below). Because various types of kinases operate in living myocardium (as discussed in Matsuba et al., 2009), future studies need to be systematically conducted to investigate whether and how these kinases affect the Ca^2+^-sensitizing effect of OM.

OM at 1.0 μM increased MgADP-induced submaximal forces in PLV, but not in PLA (+ATP and +EGTA; Figure 3). The finding in PLV supports the notion that OM apparently increases Ca^2+^ sensitivity in normal Ca^2+^-dependent contraction, independent from Ca^2+^-binding to TnC (see previous papers by others; e.g., Malik et al., 2011). There could be two reasons for the absence of the effect of OM in PLA. First, the binding affinity of OM for PLA is too low to augment submaximal forces of ADP-contraction. Second, while the OM-myosin complex effectively activates thin filaments and promotes the binding of neighboring myosin in PLV during ADP-contraction, this allosteric effect would be less in PLA due to the short attachment duration of atrial myosin. The effectiveness of MgADP in the generation of active force in ADP-contraction was consistently markedly less pronounced in PLA than in PLV (Figure 3), indicating a lesser magnitude of the effect of strongly bound cross-bridges in PLA “turning on” thin filaments. It is to be noted that OM decreased maximal force of ADP-contraction in PLV but not in PLA (Figure 3). It can be considered that due to the long attachment duration of ventricular myosin, the application of OM in the bathing solution containing 20 mM MgADP excessively promotes the formation of the non-force producing, actomyosin-ADP complex (i.e., AM.ADP2 in Fukuda et al., 1998), similar to the characteristics of rigor cross-bridges, and thereby decreases the number of force-generating, working cross-bridges.

We previously reported that the magnitude of the shift of pCa_50_ following sTn treatment depends on SL in PLV; it becomes less pronounced as SL is elongated (i.e., ∼0.23 and ∼0.12 pCa units at SL 1.9 and 2.3 μm, respectively; Terui et al., 2008). Given, therefore, that throughout the present study SL was set at 2.1 μm in both PLV and PLA, we consider that the present sTn treatment suitably increased Ca^2+^ sensitivity in both types of preparation via enhanced thin filament cooperative activation (Figure 4; cf. Terui et al., 2008). Similarly, we previously reported that pCa_50_ was shifted leftward in a SL-dependent manner in PLV upon application of 3 mM MgADP, by ∼0.26 and ∼0.13 pCa units at SL 1.9 and 2.3 μm, respectively (Terui et al., 2010). As with the case for sTn treatment, this is because the number of “recruitable” cross-bridges is decreased at a longer SL, resulting in the attenuation of further formation of force-generating cross-bridges via thin filament cooperative activation by strongly bound cross-bridges (i.e., actomyosin-ADP complex; see Fukuda et al., 1998, 2000). We therefore consider that the present finding on the MgADP-induced increase in Ca^2+^ sensitivity in PLV obtained at SL 2.1 μm (i.e., 0.24 ± 0.03 pCa units) is consistent with our previous data (Figure 4; cf. Terui et al., 2010). It should be noted that, despite the same concentration of 3 mM used, the effect of MgADP was markedly less in PLA with a leftward shift magnitude of only 0.06 ± 0.01 pCa units. We consider that the differential effect of MgADP on the leftward shift of pCa_50_ between PLV and PLA results from a difference in the apparent binding affinity of MgADP to ventricular myosin vs. atrial myosin. It has indeed been reported that the equilibrium constant of the ADP-release during the cross-bridge cycling is higher for atrial myosin than ventricular myosin (Wang et al., 2013; Walklate et al., 2021), thereby decreasing the apparent affinity of exogenously added MgADP.

Here, we discuss the possible mechanisms by which the Ca^2+^-sensitizing effect of OM was attenuated in PLV and PLA following sTn treatment and in the presence of MgADP. It should be noted that despite different approaches used, both sTn treatment and MgADP application enhance thin filament cooperative activation, as reflected by increased Ca^2+^ sensitivity, the former and latter of which act in a direct and indirect manner, respectively (see Terui et al., 2010). Recent studies have proposed a possible primary mechanism of the cardiotonic action of OM based on the prolongation of the actomyosin attachment duration and the ensuing enhancement of thin filament cooperative activation (see **Introduction**). In the present study, the magnitude of the leftward shift of pCa_50_ between sTn treatment and MgADP application was insignificant (Figure 4), suggesting that a similar magnitude of enhanced thin filament cooperative activation was attained. Likewise, the Ca^2+^-sensitizing effect of OM was insignificant between the two procedures in both PLV and PLA (Figure 4). These findings suggest that the number of “recruitable” cross-bridges is decreased by a similar magnitude following sTn treatment and MgADP application, thereby attenuating the effect of OM to recruit neighboring myosin into the force-generating state via enhancement of thin filament cooperative activation.

The depressant effect of Pi on the OM-induced increase in Ca^2+^ sensitivity was evident in both PLV and PLA (Figure 5). The present finding on PLV is consistent with our previous result in that pCa_50_ was shifted rightward in a SL-dependent manner, by ∼0.27 and ∼0.20 pCa units at SL 1.9 and 2.3 μm, respectively (cf. Terui et al., 2010). We found in the present study that the Ca^2+^-sensitizing effect of OM was markedly decreased in the presence of 20 mM Pi in both PLV and PLA (Figure 5). It has been reported that Pi binds to the actomyosin-ADP complex after the power stroke in the cross-bridge cycle and causes reversal of the stroke (Woody et al., 2019), and accordingly, Ca^2+^ sensitivity is apparently decreased (see Fukuda et al., 1998 and references therein). We therefore consider that although the number of “recruitable” cross-bridges is increased in the presence of Pi (Terui et al., 2010), regardless of the type of preparation (PLV or PLA), due to the acceleration of detachment of OM-bound myosin from thin filaments, thin filament cooperative activation is depressed; consequently, the number of cross-bridges recruited to thin filaments will be limited. Nevertheless, the cardiotonic action of OM was seen under this depressed condition to increase maximal force in PLA (Table 4), which was not the case under enhanced thin filament activation conditions, regardless of the type of preparations (Table 3).

It should be noted that the Ca^2+^-sensitizing effects of OM may be coupled with a change in the thick filament state, independent of the thin filament “*on-off*” regulation. Stewart et al. (2010) discovered a myosin state in skinned rabbit psoas muscle fibers that has an extremely slow ATP turnover rate. This unique relaxed state has come to be widely known as the “super-relaxed state” (SRX) (see reviews and references therein: e.g., Cooke, 2011; McNamara et al., 2015; Irving, 2017; Spudich, 2019; Craig and Padrón, 2022). SRX is reportedly in equilibrium with the disordered-relaxed state (DRX) in which myosin heads are in closer proximity to thin filaments (e.g., Anderson et al., 2018; Gollapudi et al., 2021; Yuan et al., 2022), and ready to produce force. A decrease in the number of myosin molecules in SRX would increase the proportion of the heads available to form force-generating cross-bridges (e.g., Schmid and Toepfer, 2021). It is possible that mechanical stress on thick filaments, presumably via myosin binding to thin filaments, shifts the SRX-DRX equilibrium towards DRX (Linari et al., 2015). It is considered that SRX is a biochemical and presumably a structural state, in which myosin heads interact with, and are folded back on, the thick filament backbone, and are consequently unavailable for binding thin filaments (e.g., Hooijman et al., 2011; Alamo et al., 2016). Hooijman et al. (2011) reported that in cardiac muscle, a significant proportion of myosin in thick filaments is in SRX, and these myosin molecules in SRX are rather modulatory, providing more graded recruitment of the heads and the ensuing active force production, compared with skeletal muscle. Therefore, OM may modulate the thick filament structure and thereby destabilize SRX, resulting in an increase in the number of myosin heads that can readily bind to thin filaments and produce active force in a graded manner (Kampourakis et al., 2018). We consider that this thick filament-based mechanism may at least in part account for the observed Ca^2+^-sensitizing effects of OM in both PLV and PLA (Figure 1). It is likewise possible that OM’s effect is synergistically brought about via the thick filament-based regulation and the ensuing thin filament “*on-off*” regulation (Spudich, 2019; Nag and Trivedi, 2021). Namely, myosin in the pre-pre-powerstroke state, a state similar to SRX, is stabilized by OM, but not sufficiently compliant to fit into the folded-back thick-filament state and therefore it is able to bind to actin. Ca^2+^ sensitivity would consequently be increased via formation of myosin in the pre-pre-powerstroke state and the ensuing thin filament cooperative activation, as well as by a reduction in the number of myosin heads in the folded-back state.

Likewise, it is important to discuss the possible contribution of the thick filament-based SRX-DRX regulation to the attenuation of the Ca^2+^-sensitizing effect of OM. First, PKA-dependent phosphorylation of cardiac MyBP-C has been reported to shift the SRX-DRX equilibrium to DRX (McNamara et al., 2019; Harris, 2021). Therefore, fewer cross-bridges would be recruited by OM when MyBP-C is phosphorylated, resulting in the attenuation of OM’s Ca^2+^-sensitizing effect in both PLV and PLA (Figure 2). Along this line, it is possible that MgADP shifts the SRX-DRX equilibrium to DRX, via structural changes in thick filaments (see Nag and Trivedi, 2021 and references therein), reduces the number of “recruitable” cross-bridges, and thereby attenuates OM’s effect (Figure 4). Future studies are needed from the perspective of thick filaments by using various techniques to determine whether and how OM’s effect varies in ventricular and atrial muscles under various perturbations.

Ovejero et al. (2022), by taking advantage of X-ray diffraction, reported that upon a decrease in temperature from 39°C to 7°C, myosin heads leave the helical folded state of thick filaments, and the periodicity of the thick filament backbone increases in intact rat ventricular trabeculae. They likewise demonstrated that myosin filament structure and motor conformation of intact trabeculae at 39°C are largely preserved in skinned trabeculae at 27°C or above in the presence of osmotic compression. However, when active force levels are high at high temperatures (e.g., 27°C), or at longer sarcomere length, rundowns easily occur during force measurements in mechanical experimentations with skinned muscle preparations, which would lead to an underestimation of maximal active force and could therefore impact the deduced Ca^2+^ sensitivity (as discussed in Fukuda et al., 2005b). The results of the present study obtained at a low temperature of 15°C might therefore lead to an underestimation of the Ca^2+^-sensitizing effect of OM because of a loss of cross-bridges in SRX in the relaxing condition that can potentially be activated by OM and form into force-generating cross-bridges.

Finally, we discuss the clinical relevance obtained from the present findings. First, OM increased Ca^2+^ sensitivity at clinically relevant concentrations in PLV and PLA, with the effect more pronounced in PLV. Careful studies are needed to investigate whether OM increases ventricular contraction to a greater magnitude than atrial contraction *in vivo*, especially in patients with HFrEF, thereby effectively increasing cardiac output without causing congestion. Second, the greater Ca^2+^-sensitizing effect in PLV was retained following PKA treatment, suggesting that OM may be a useful cardiotonic compound under the various PKA-dependent phosphorylation states of myofibrillar proteins. Third, given that OM’s Ca^2+^-sensitizing effect became diminished following sTn treatment in both types of preparations, its cardiotonic effect may be less in patients with HFrEF associated with Tn mutations. Fourth and finally, the marked attenuation of OM’s Ca^2+^-sensitizing effect in the presence of Pi in both PLV and PLA suggests that the compound’s cardiotonic effect may be limited in the ischemic heart or under hypoxia. Future basic and clinical studies are warranted to systematically investigate whether and how the inotropic effect of OM is altered in the ventricle and atrium under various disease conditions of the heart.

## Data Availability

The raw data supporting the conclusions of this article will be made available by the authors, without undue reservation.

## Abbreviations

BDM, 2,3-butanedione 2-monoxime; CP, phosphocreatine; CPK, creatine phosphokinase; DRX, disordered-relaxed state; DTT, dithiothreitol; HF, heart failure; HFrEF, heart failure with reduced ejection fraction; MyBP-C, myosin-binding protein C; OM, omecamtiv mecarbil; Pi, inorganic phosphate; PKA, protein kinase A; PLA, porcine left atrial muscle; PLV, porcine left ventricular muscle; SL, sarcomere length; SRX, super-relaxed state; sTn, fast skeletal troponin complex.
